# Chimpanzee (*Pan troglodytes*) Precentral Corticospinal System Asymmetry and Handedness: A Diffusion Magnetic Resonance Imaging Study

**DOI:** 10.1371/journal.pone.0012886

**Published:** 2010-09-21

**Authors:** Longchuan Li, Todd M. Preuss, James K. Rilling, William D. Hopkins, Matthew F. Glasser, Bhargav Kumar, Roger Nana, Xiaodong Zhang, Xiaoping Hu

**Affiliations:** 1 Biomedical Imaging Technology Center, Emory University, Atlanta, Georgia, United States of America; 2 Division of Neuroscience, Yerkes National Primate Research Center, Atlanta, Georgia, United States of America; 3 Department of Pathology, Emory University, Atlanta, Georgia, United States of America; 4 Center for Behavioral Neuroscience, Emory University, Atlanta, Georgia, United States of America; 5 Division of Psychobiology, Yerkes National Primate Research Center, Atlanta, Georgia, United States of America; 6 Department of Anthropology, Emory University, Atlanta, Georgia, United States of America; 7 Department of Psychiatry and Behavioral Sciences, Emory University, Atlanta, Georgia, United States of America; 8 Department of Psychology, Agnes Scott College, Decatur, Georgia, United States of America; 9 Department of Biomedical Engineering, Georgia Institute of Technology/Emory University, Atlanta, Georgia, United States of America; University of Minnesota, United States of America

## Abstract

**Background:**

Most humans are right handed, and most humans exhibit left-right asymmetries of the precentral corticospinal system. Recent studies indicate that chimpanzees also show a population-level right-handed bias, although it is less strong than in humans.

**Methodology/Principal Findings:**

We used *in vivo* diffusion-weighted and T1-weighted magnetic resonance imaging (MRI) to study the relationship between the corticospinal tract (CST) and handedness in 36 adult female chimpanzees. Chimpanzees exhibited a hemispheric bias in fractional anisotropy (FA, left>right) and mean diffusivity (MD, right>left) of the CST, and the left CST was centered more posteriorly than the right. Handedness correlated with central sulcus depth, but not with FA or MD.

**Conclusions/Significance:**

These anatomical results are qualitatively similar to those reported in humans, despite the differences in handedness. The existence of a left>right FA, right>left MD bias in the corticospinal tract that does not correlate with handedness, a result also reported in some human studies, suggests that at least some of the structural asymmetries of the corticospinal system are not exclusively related to laterality of hand preference.

## Introduction

One of the most robust behavioral manifestations of hemispheric specialization in humans is handedness [1], [2], [3]. Although there is some cultural variation, a significant majority of humans prefer to use the right hand for a number of different actions [4], [5], [6]. For example, studies by Annett indicate that approximately 64% of humans consistently use the right hand for all self-reported actions, with 3 – 4% consistently using the left hand for all actions and ∼30% using the right hand for some actions and left hand for others [7], [8].

Historically, it has been argued that population-level handedness is unique to humans and emerged after the split between the chimpanzee and human lineages [9], [10]. Moreover, many have claimed that the evolution of right-handedness in humans was associated with the emergence of higher-order complex cognitive and motor processes such as language and speech as well as tool use [4], [11], [12]. Recently, the claims of human-specific population-level handedness have been called into question based on studies in nonhuman species, notably nonhuman primates and particularly our closest living relatives, the chimpanzees [13], [14]. Population-level right handedness in chimpanzees has been reported for a number of behaviors [15], [16] and observed in both captive and wild chimpanzees [17], [18], [19], [20], [21], [22], although the bias toward right-handedness is less strong than in humans. For example, in a coordinated bimanual task, in which peanut butter was smeared on the inside edge of polyvinyl-chloride tubes and the hand used by an individual to extract the peanut butter was recorded for over 100 chimpanzees, 51 – 56% of chimpanzees consistently used the right hand for the action, 27 – 32% used the left hand, and 14 – 19% were classified as ambidextrous [23], [24], [25], [26].

One question that emerges from the behavioral studies of handedness in chimpanzees is whether similar neurological mechanisms underlie the expression of handedness in both chimpanzees and humans. Laterality of hand preference is expected to be reflected in anatomical asymmetries, and a number of studies in humans have indicated the existence of interhemispheric asymmetries in the primary motor cortex in humans as well as evidence of a relationship between asymmetries and handedness [27], [28], [29], [30]. For example, humans exhibit differences in the morphology of the central sulcus, which is deeper in the left hemisphere of right handers and in the right hemisphere of left handers [27], although in a subsequent study with a larger sample the handedness effect was only demonstrated in human males [28].

While T1-weighted MRI can be used to study sulcal morphology and tissue volume, the use of diffusion MRI makes it possible to explore asymmetries of white-matter organization [31], [32]. Diffusion MRI is an *in vivo* imaging technique that can be used to characterize the magnitude and direction of water molecule diffusion [33], [34]. One way to characterize water diffusion is in terms of fractional anisotropy (FA), which is a scalar measure of the degree of anisotropic water diffusion at a voxel [33], [35], and of mean diffusivity (MD), which characterizes the overall displacement of molecules (average ellipsoid size) and the overall presence of obstacles to diffusion. Variations in FA and MD are thought to relate to the microstructural features of white-matter organization, such as the amount of myelination and the density, diameter, and directional coherence of axons [29]. Diffusion MRI also allows the three-dimensional trajectories of fiber bundles in the white matter to be reconstructed by piecing together discrete estimates of the underlying continuous fiber-orientation fields, making noninvasive study of brain connectivity possible [36], [37], [38], [39]. Diffusion MRI has been used to study motor-related interhemispheric white-matter differences in humans [29], [40], [41], [42], [43], [44]. For example, Buchel et al. used a voxel-based morphometry (VBM) approach to study the relationship between hemispheric FA differences and handedness, and found a leftward bias (left>right) in FA of the white matter of the precentral gyrus for right-handers and *vice versa* for left handers; that is, a hemisphere-by-handedness interaction [29]. Two other studies of the relationship between corticospinal FA and handedness, however, using region-of-interest (ROI) [43] and tractography [40] methods, revealed only a hemispheric asymmetry (left>right FA) but no correlation of asymmetry with handedness.

To date, the relationship between cerebral asymmetries and handedness in chimpanzees has been studied only with T1-weighted images, involving mainly ROI analyses of gray- and white-matter volumes and morphometrics of the central sulcus [45], [46], [47], [48]. These results are similar to those that have been reported in humans. For example, Hopkins and Cantalupo [45] reported an interaction between hand preference and the volume of the hand representation of primary motor cortex (the “knob” region), and Dadda et al. [46] reported an interaction between hand preference and the depth of the central sulcus at the level of the hand representation.

In the present study, we used diffusion-weighted MRI, along with T1-weighted images, to examine the microstructural and macrostructural asymmetries of the corticospinal system and determine their relationship to handedness in 36 adult female chimpanzees. We investigated interhemispheric differences in the precentral corticospinal system at the microstructural level, as quantified by FA and MD, and their relationship to handedness, analyzing the diffusion data in several different ways. Analyses included a voxel-wise approach using tract-based spatial statistics (TBSS), a region-of-interest (ROI) analysis based on the white-matter skeleton derived from TBSS, and an analysis of FA and MD values within the corticospinal tract as delineated by tractography. TBSS is a novel group analysis method that is expected to yield better alignment of FA maps from different subjects compared to the conventional VBM-style registration, and thus improved statistical detection of FA differences. TBSS yields a skeleton of white matter voxels that can be analyzed in a voxel-wise manner (conceptually similar to voxel-based morphometry; VBM), or by using an ROI approach, which mitigates the problems of partial voluming and the multiple-comparison corrections required when statistically comparing the large numbers of voxels involved in a voxel-based analysis. In a third approach, we used probabilistic tractography to identify and delineate the precentral corticospinal tract within the white matter, and then analyzed FA and MD within voxels assigned to the CST at different levels of the tract. By delineating the CST, it was also possible for us to examine interhemispheric asymmetries in CST macrostructure, specifically, its anatomical location within the white matter of chimpanzees. Finally, we determined the depths of the central sulci (CS) of each chimpanzee on T1-weighted images and correlated CS depth with handedness and with FA/MD from within the precentral corticospinal tracts. Based on the previous reports on the relationships of human corticospinal tract asymmetries and handedness [40], [43], we hypothesized that, as reported in humans, chimpanzees' corticospinal tract asymmetries should be independent of handedness. We also hypothesized that since CS depth in both chimpanzees and humans has been reported to correlate with handedness, a handedness effect on CS depth should be observed in our samples. In addition, we would expect asymmetries of CS depth and of corticospinal tract microstructure (i.e., FA and MD) to be uncorrelated.

## Methods

### Subjects

MRI scans were obtained from 36 adult female chimpanzees. All chimpanzees were members of a colony at Yerkes National Primate Research Center (YNPRC) in Atlanta, Georgia. The individual characteristics of the chimpanzees are summarized in Table 1. The animals used in this study are part of an ongoing study that compares brain aging in adult females of several primate species. Possible relationships between DTI-derived indices of microstructure and age will be explored in a separate paper, currently in preparation. We do not currently have access to a comparable set of MRI scans from male chimpanzees.

**Table 1:** Subject characteristics of the 36 chimpanzees.

		Handedness on TUBE task			
	Subjects(n = 36)	Right-handed(n = 18)	Non-right-handed(n = 18)		Statistics^a^
			Mixed-handed	Left-handed	
**Age at imaging-years, M(±SD)**	26.72(±13.24)	26.06(±12.93)	19.17(±8.18)	31.50(±14.56)	F_(1,34)_ = 0.089, p<0.77
**Weight at imaging-kg, M(±SD)**	64.74(±13.39)	65.99(±8.38)	65.58(±12.58)	62.43(±19.53)	Z_(1,34)_ = −0.554, p<0.58
**Brain Volumes-cm^3^, M(±SD)**	391(±30)	389(±29)	409(±38)	387(±28)	F_(1,34)_ = 0.220, p<0.64
**TUBE z-scores**	0.57(±6.35)	5.70(±2.64)	−0.11(±1.50)	−6.76(±3.80)	F_(1,34)_ = 8.304, p<1×10^−9^

Note: a) As the sample of mixed-handed subjects was small (n = 6), we classified all of the subjects with z scores less than 1.95 as non-right-handed and the statistics were based on the right-handed group and non-right-handed group.

### Procedure and acquisition of magnetic resonance images

All studies were conducted at the YNPRC of Emory University. Both YNPRC and Emory University are accredited by the Association for Assessment and Accreditation of Laboratory Animal Care (AAALAC) International. The procedures described for husbandry were in accord with guidelines of YNPRC and the protocol described for obtaining MRI scans was approved by the Institutional Animal Care and Use Committee (IACUC, approval number: 194-2009Y) of Emory University.

Prior to scanning, the subjects were immobilized with ketamine injections (2−6 mg/kg, i.m.) and were subsequently anesthetized with an intravenous propofol drip (10 mg/kg/hr) following standard veterinary procedures used at YNPRC. The subjects remained sedated for the duration of the scans as well as the time needed for transport between their home cage and the scanner location. After completing the MRI scan, the chimpanzees were temporarily housed in a single cage for 6 to 12 hours to allow the effects of anesthesia to wear off before being returned to their home cage and cage mates. The veterinary staff and research staff observed the general well-being (i.e., activity, food intake) of the chimpanzees twice daily after the scan for possible distress associated with anesthetic accesses.

Both anatomical and diffusion MRI was performed on a Siemens 3T Trio scanner (Siemens Medical System, Malvern, PA) with a standard birdcage coil. Foam cushions and elastic straps were used to minimize head motion. High-resolution T1-weighted images were acquired with a 3D magnetization-prepared rapid gradient-echo (MPRAGE) sequence for all participants. The scan protocol, optimized at 3T, used a repetition time/inversion time/echo time of 2400/1100/4.13 msec, a flip angle of 8°, a volume of view of 256×256×154 mm, a matrix of 256×256×192, and resolution of 0.8×0.8×0.8 mm, with 2 averages. The orientations of the 60 diffusion directions are plotted in Fig. S1. Total T1 scan time was approximately 20 minutes.

Diffusion MRI data were collected with a diffusion-weighted, multi-shot (four segments), spin-echo echo planar imaging (EPI) sequence. A dual spin-echo technique combined with bipolar gradients was used to minimize eddy-current effects [49]. The parameters used for diffusion data acquisition were as follows: diffusion-weighting gradients applied in 60 directions with a b value of 1000 sec/mm^2^; repetition time/echo time of 5740/91 msec, field of view of 230×230 mm^2^, matrix size of 128×128, resolution of 1.8×1.8×1.8 mm, 41 slices with no gap, covering the whole brain. Averages of two sets of diffusion-weighted images with phase-encoding directions of opposite polarity (left – right) were acquired to correct for susceptibility distortion [50]. For each average of diffusion-weighted images, six images without diffusion weighting (b = 0 sec/mm^2^) were also acquired with matching imaging parameters. The total diffusion MRI scan time was approximately 50 minutes.

### Behavioral measure of handedness

The handedness of the subjects was assessed with a task that measures coordinated bimanual actions, referred to as the TUBE task [23]. This measure of handedness was selected for several pragmatic and scientific reasons. First, handedness data on the TUBE task were available for all subjects in this study. Second, previous studies in chimpanzees have shown that handedness for TUBE task are stable across time with test-retest r values exceeding 0.66 over a 6-year test period [24]. Furthermore, the TUBE task elicits strong individual hand preferences, with very few individuals showing ambiguous hand preferences, likely due to the fact that hand use for this task is relatively immune to situational factors. Third, hand preference data on the TUBE task have been collected in more than 500 chimpanzees residing in 3 different research laboratories and the evidence of population-level right handedness is consistent across all 3 samples [51]. Thus, the TUBE task is a valid and reliable measure of handedness. Finally, previous studies have shown that variation in hand preference for the TUBE are associated with asymmetries in the portion of the precentral gyrus representing the hand, not only in chimpanzees (see above) but also in at least one report in capuchin monkeys [52].

Briefly, in the TUBE task, peanut butter was smeared on the inside edges of polyvinyl chloride (PVC) tubes approximately 15 cm in length and 2.5 cm in diameter. The peanut butter was smeared on both ends of the PVC pipe and was placed far enough down the tube so that the subjects could not lick the contents completely off with their mouths but had to use one hand to hold the tube and the other hand to remove the peanut butter. The PVC tubes were handed to the subjects in their home cages and a focal sampling technique was used to collect data from each subject. Each time the subjects reached into the tube with their finger, extracted peanut butter and brought it to their mouth, the hand of the finger used to extract the peanut butter was recorded as left or right. A minimum of 50 responses were obtained from each subject.

For each subject, a handedness index (HI) was determined using the formula HI  =  (R – L)/(R + L) where R and L represent the frequency of left and right hand use on the TUBE task. HI values varied from −1.0 to 1.0 with negative values reflecting left-hand biases and positive values reflecting right-hand biases. In addition, a *z*-score was derived for each subject based on the frequency of left- and right-hand use. Subjects with *z* scores greater than 1.95 or less than −1.95 were classified as right- and left-handed, respectively. Subjects with *z* scores between −1.95 and 1.95 were classified as mixed-handed. Based on this criterion, 18 (50%) out of the 36 chimpanzees were classified as right-handed, 12 (33%) were classified as left-handed and 6 (17%) as mixed-handed, consistent with hand distribution in previous studies [23], [24], [25], [26]. Because the number of mixed-handed subjects was small (n = 6), we classified all of the subjects with z scores less than 1.95 as non-right-handed [53].

### Image Processing

Both anatomical and diffusion MR data were analyzed using tools from the Oxford Center for Functional Magnetic Resonance Imaging of the Brain's software library (FSL, http://www.fmrib.ox.ac.uk/fsl/). T1-weighted images were preprocessed with skull stripping [54], intensity bias correction [55], noise reduction [56], and contrast enhancement (squaring the images and then dividing by the mean). Diffusion MR data were first corrected for eddy-current distortion and for susceptibility distortion following the method of Andersson et al. [50] using Matlab (Matlab7, Mathworks) codes incorporated in SPM5 (http://www.fil.ion.ucl.ac.uk/spm/).

### Voxelwise analysis using TBSS

**Figure 1:**
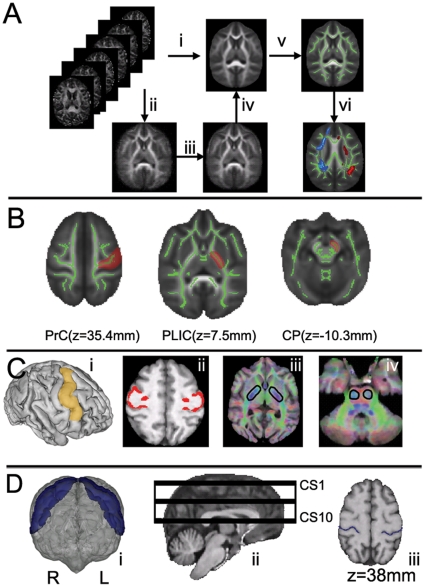
Procedures for TBSS, masks in tractography, and measurement of CS depth. **A**: Image registration procedure for TBSS: All subjects' FA images were registered on a cohort-specific template (A.i.). A three-stage procedure was used for generating the cohort-specific FA template. First, a randomly selected subject's FA map was aligned so that the midsagittal plane was defined by the interhemispheric fissure of the brain, the axial plane was situated parallel to the bicommissural line (anterior commissure and posterior commissure), and the origin of the space was defined as the center of the anterior commissure. Then, all of the other FA maps were aligned to the first FA map using rigid body registration with six degrees of freedom (A.ii.). The individually aligned FA maps were then averaged to generate a template with six degrees of freedom. Second, the FA template (6 degrees of freedom) was employed as the initial FA template on which each subject's FA map was registered using twelve degrees of freedom affine registration (A.iii.). These were then averaged to create an intermediate FA template (12 degrees of freedom) for the final alignment of the FA maps using nonlinear registration (A.iv.). This population-specific FA template obtained with nonlinear registration was then used as the template for TBSS registration. Then, a symmetrical FA skeleton for each FA image was derived representing the centers of all tracts common to all the chimpanzees (A.v.). FA and FA difference maps derived from the symmetrical FA skeleton were then evaluated with permutation-based statistics to test for left-right differences (A.vi.). **B**: The locations of the three ROIs in the skeleton-based ROI analysis. **C**: The cortical mask, waypoint mask and seed mask used in tractography for deriving the precentral corticospinal tract. The schematic in C.i. shows the coverage of the precentral GM/WM boundary cortical masks. C.ii. provides a 2D view of the precentral GM/WM boundary cortical masks at an axial slice, while C.iii. shows the waypoint mask at the posterior limb of internal capsule, and C.iv. shows the seed mask at the level of the pons. **D**: The schematic of the depth of the CS. The schematic of the CS in a 3D view (D.i.); the intrasulcal length of the posterior contour of the precentral gyrus was traced in a dorso-ventral sequence of 45 slices extending from z = 9∼45 mm covering the primary motor cortex, which was subsequently divided into ten evenly spaced subsections from the most ventral region (CS1) to the most dorsal region (CS10) (D.ii.). The horizontal black bands illustrates the relatively volume covered in each subregion. An example slice at z = 38 mm was shown in D.iii., with the traced depth of the CS denoted as blue color.

Voxelwise statistical analysis of the diffusion MR data was carried out using TBSS implemented in FSL 4.1 (FMRIB, Oxford. http://www.fmrib.ox.ac.uk/fsl/). The reason that TBSS was chosen over other methods for analyzing group differences, such as a VBM-style approach [29], is because it has been shown to be able to resolve the alignment issues of FA images from multiple subjects and does not require arbitrarily chosen smoothing kernels for valid statistics [57], [58]. This method has been used in studies on healthy subjects [59] as well as in pathological populations [60], [61], and is considered a more sensitive tool for voxelwise analysis of white-matter alterations than a VBM-style approach [57], [62]. The standard procedures in TBSS were followed (Fig. 1A). All subjects' FA images were aligned to a population-specific FA template derived from the full set of subjects, using a non-linear registration algorithm, FNIRT, implemented in the FSL package. These individual FA images were then averaged to create a mean FA image, which was thinned to generate an FA skeleton that represents the centers of tracts common to all individuals. The FA map from each subject was thresholded at FA>0.3 so as to include major white-matter pathways but exclude minor tracts in which there was substantial inter-subject variability and/or partial volume effects. Subsequently, this skeleton was left/right flipped and averaged to make it symmetrical, and each subject's FA map was then projected onto the symmetrical skeleton, by filling the skeleton with FA values from the nearest relevant tract centre. This was achieved, for each skeleton voxel, by searching the surface perpendicular to the local skeleton structure for the maximum FA value in the subject's FA image [57]. An FA difference map (L– R), as well as an asymmetry quotient (AQ) map, with AQ  =  (L – R)/(L + R), where L and R represent the FA values in the left and right hemispheres, respectively, were generated to investigate the main effects of hemisphere and handedness and their interaction (see *Statistical Analysi*s, below, for details).

### Template Creation

To align the FA images of all subjects to a common space for comparison, a population-specific FA template was created using a three-stage procedure (Fig. 1A). First, the FA images from a randomly selected subject were aligned so that the sagittal plane was parallel to the interhemispheric fissure of the brain, and the axial plane was perpendicular to the sagittal plane and parallel to the bicommissural line (the line connecting the anterior and posterior commissures) [63]. The origin of the space was defined as the center of the anterior commissure. Then, all the other FA maps were aligned to the first FA map using rigid-body registration with six degrees of freedom. The set of aligned FA maps was then averaged to generate an initial template. Each subject's FA map was then registered to this initial FA template using an affine registration with 12 degrees of freedom. These individually aligned FA maps were subsequently averaged to create an intermediate FA template (12 degrees of freedom) for the final alignment of the FA maps using FNIRT nonlinear registration. The resulting population-specific FA template was used as the template for TBSS registration. A similar procedure was applied on the T1-weighted maps for generating a population-specific T1 template.

### Skeleton-based ROI method

The reasons for employing the skeleton-based ROI method are twofold: first, voxelwise whole-brain analysis involves multiple voxels (order of 10^6^ in the current study) and stringent corrections for multiple comparisons are required for identifying significant effects. The ROI method, on the other hand, does not require such a correction and should therefore be more sensitive for detecting differences within selected ROIs. Second, partial voluming is considerable, even in our diffusion MRI data with the resolution of 1.8 mm^3^ isotropic, likely due to chimpanzee's smaller brain volume compared to humans. Comparing only the maximal FA values projected onto the white-matter skeleton within the ROIs (i.e., the skeleton-based ROIs) could minimize the partial volume issue. As the major focus of ROI analysis in this study was on the precentral corticospinal system, three ROIs—in the white matter of the precentral gyrus (PrG), the posterior limb of the internal capsule (PLIC), and the cerebral peduncle (CP)—were manually drawn on four consecutive axial slices of the skeletonized FA map (see Fig. 1B). To avoid possible bias in selecting ROIs, the following objective criteria were used. For the PrG ROI, a mask was drawn on four consecutive axial slices approximately spanning the “hand knob” (z = 35.4 mm) [45]. This area was chosen because it is known to be involved in motor-related tasks and has been a focus of anatomical studies in chimpanzees. For the PLIC ROI, the mask was drawn at z = 7.5 mm, where the PLIC could be clearly seen on both FA maps and eigenvector color maps. The CP ROI began superiorly in an axial slice where the optic tract and the cerebral peduncle start to separate (z = −10.3 mm) and extended inferiorly for four consecutive slices. The same procedure was carried out for the MD, axial diffusivity (AD, λ1) and radial diffusivity (RD, (λ2+ λ3)/2) skeleton maps of the 36 subjects, and the mean FA, MD, AD and RD values in the skeleton defined by the three ROIs in each hemisphere were calculated and compared.

### Probabilistic fiber tracking of corticospinal tracts

Probabilistic tractography, as implemented in FSL, was used to track the corticospinal tracts originating from the precentral gyrus. We used a seed mask at the middle level of the basilar pons and a cortical target mask at the precentral gyrus, with a waypoint mask at the posterior limb of internal capsule, a structure through which corticospinal fibers pass [64], [65], [66]. We repeated the tractography analysis on four chimpanzees in our samples with the seed mask located at a more inferior level of the basilar pons, in order to determine whether seed masks placed at mid-pontine levels yield tracts that include corticopontine as well as corticospinal fibers.

The procedures used for generating seed masks, waypoint masks, and cortical target masks were as follows. All cortical target masks were derived from the T1-weighted images. The waypoint masks and seed masks were drawn on the color map of the diffusion MRI data transformed to each subject's T1 space using a rigid-body transformation. After this transformation, there was no discernable mismatch between our susceptibility distortion-corrected diffusion MR data and its corresponding T1-weighted image. Both the seed and waypoint masks were drawn on four consecutive axial slices in each subject's original T1-weighted map. For the gray-matter/white-matter (GM/WM) boundary cortical masks of the precentral gyrus, the T1-weighted map for each subject was segmented into WM, GM, and cerebrospinal fluid (CSF), and their partial volume estimates also obtained [55]. The partial volume estimates of the GM and WM were dilated, thresholded (> 0.6), and binarized. The overlap of the processed GM and WM probability maps was defined as the GM/WM boundary mask, and had a thickness of approximately 2 mm. Next, two initial ROIs intended to include the left and right primary motor cortices were drawn by hand on the T1 template. The initial ROIs included the precentral gyrus laterally and paracentral lobule medially, with the inferior border on the lateral surface at approximately the same horizontal level as the upper end of the Sylvian fissure (Fig. 1C), a border chosen to match the inferolateral extent of Brodmann's area 4 as previously mapped in humans and chimpanzees [67], [68], [69]. The initial precentral ROIs may include part of the ventral premotor cortex as well as primary motor cortex, according to the chimpanzee map of Bailey et al. [69]. The two initial ROIs were nonlinearly transformed back to each individual subject's T1-weighted image. A Boolean AND operation was performed between these ROIs of the precentral gyrus and the GM/WM boundary mask to derive a GM/WM boundary cortical mask of the precentral gyrus in each subject (see Fig. 1C). We also made an exclusion mask along the midline of the brainstem, because corticospinal fibers are believed to be restricted largely or exclusively to the hemisphere of origin until they reach the decussation at the junction of the medulla and spinal cord.

For each subject, probabilistic tractography was carried out from all voxels in each seed mask for each hemisphere, using a model that accommodates two fiber orientations [39]. Probability density functions were calculated by drawing 20,000 samples from the uncertainty distribution of the fiber orientations between the seed mask at the level of pons and the cortical target mask. The number of these samples that passed through the waypoint mask at the PLIC and reached the precentral gyrus GM/WM boundary cortical target mask was counted as the waytotal. After each subject's precentral corticospinal tracts had been tracked, we normalized the corticospinal pathways by waytotal number, and the normalized value on each voxel was interpreted as the tract probability given the prior knowledge that the tract exists on that voxel. Then, each subject's tract probability of the precentral corticospinal tract was transformed to the T1 template using nonlinear transformation as described above. Finally, all these transformed precentral corticospinal tracts in the common space were averaged and multiplied by 100 to generate a percentile track probability for the precentral corticospinal tract in this population. The population-level tract probability that voxel A and B are connected, P(A→B), was obtained using the following formula:
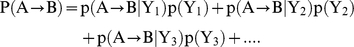
where Y_1_, Y_2_, … ,Y_i_ are the data from the different subjects in this population, which are equally probable a priori: p(Y_1_) = p(Y_2_) = … = p(Y_i_).

### Tract-based ROI analysis

An alternative to defining white-matter ROIs or using voxelwise TBSS analysis to examine the whole brain white matter is to use diffusion tractography to estimate the course of fiber pathways through the white matter and then derive measures such as FA from within those pathways. After the tract probability for the population was derived, it was thresholded at 0.2% and then binarized to form a tract-based mask. Several other thresholds, specifically, 0.1% and 0.07%, were also tested and they yielded asymmetry result consistent with the 0.2% threshold. To avoid biasing the estimate of the average FA toward low values due to partial voluming, each subject's FA map was used as a mask to include only those voxels with FA larger than 0.3. Then, the mean FA within this tract-based corticospinal mask was calculated based on each subject's FA map in the common FA space. We further divided the corticospinal tracts mask (thresholded at 0.2%) into five evenly spaced sections along the inferior-superior axis with z ranging from 70 to 150 mm. The mean FA values within the five sections were calculated separately and compared across hemispheres. To investigate whether the possible asymmetry in FA was related to the asymmetry in corticospinal tract volume, the tract volume with the 0.2% threshold was also collected for analysis. The same procedure was duplicated for the MD, AD, and RD maps of the 36 chimpanzees.

### Measuring the depth of the central sulcus

The T1-weighted images from the 36 chimpanzees were first aligned to the cohort-specific chimpanzee template using rigid-body registration with six degrees of freedom (translating and rotating). Then, the depth of the central sulcus was measured in serial 0.8 mm axial T1-weighted images, consistent with procedures used by others [28], [46]. For each hemisphere, the CS was traced from the crown of the anterior bank to its fundus, in a superior-to-inferior sequence of 40 axial slices extending from z =  49 to z = 9 mm (see Fig. 1D). Two analyses were conducted to investigate hemispheric and handedness effects. First, the region was divided into ten evenly spaced sectors from the most superior level (CS1) to the most inferior level (CS10). Second, the depths of the ten sectors were summed to represent the total depth of the CS. Individual AQ was derived for each sector and for the total depth of the CS using the formula AQ  =  (L – R)/(L + R), where L and R represent the depth of the CS in the left hemisphere and right hemisphere, respectively, in each sector. A subset of ten subjects from our cohort was randomly selected for checking inter-rater variability (L.L. and B.K.) and the handedness of the ten subjects were blinded to the raters; the inter-rater correlations coefficients for the left and right central sulci depths were 0.918 and 0.897 respectively.

### Statistical analysis of asymmetry and handedness

To evaluate asymmetries in the TBSS analysis, we first conducted a permutation-based one-sample *t*-test on FA difference maps to test for an effect of hemisphere. Second, we correlated AQ with HI. Third, we calculated binomial *z* scores for each subject to assess handedness based on the frequency of right-hand and non-right-hand use. We then used two-sample unpaired *t*-tests on the original FA maps to determine if there was a handedness effect, and on the FA difference maps to test for a hemisphere-by-handedness interaction. The effects of hemisphere and handedness were modeled separately because Randomize, the permutation program in the current version of FSL (v4.1), cannot model within-subject and between-subject factors together. Five thousand random permutations were generated to derive the null distribution. The family-wise error (FWE) rate was controlled using threshold-free cluster enhancement [70] and is exact for the test (set at p_FWE_ <0.01), meaning the probability of one or more type I errors is the same as the significance level [71].

Possible effects of hemisphere and handedness on the mean FA, MD defined by skeleton- and tract-based ROI analyses as well as the depth of the central sulcus were evaluated with two-way MANOVA tests, with handedness as the between-subject factor and hemisphere (left vs. right) as the within-subject factor, followed by *post-hoc* univariate tests, using SPSS 17.0 (SPSS Inc, Chicago, IL, USA). Nonparametric Spearman rank-order correlation was used to test the association of the AQ of each ROI with the HI. The effect size was determined as the proportion of variance explained (partial eta squared). The power analysis shows that with the current sample size (n = 36), the population effect size is 0.56 with the statistical power of 0.90 (α = 0.05, two-tailed testing).

## Results

### Voxelwise analysis using TBSS

**Figure 2:**
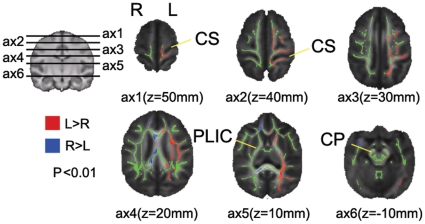
TBSS analysis of the relationship between hemisphere and FA in the precentral corticospinal system. The figurine on the left shows the positions of the six axial slices. FA statistical maps are superimposed on the mean FA skeletons, voxels shaded green are those for which FA exceeds threshold (FA>0.3). The colors superimposed on these maps indicate FA asymmetries, with red denoting left>right FA and blue denoting right>left FA. The red and blue pixels are enlarged for emphasis. Extensive left>right FA was found at the precentral gyrus. No right>left FA that is relevant to the corticospinal system was detected. CS: central sulcus; PLIC: posterior limb of internal capsule; CP: cerebral peduncle.

The results of the TBSS analysis on FA are shown in Fig. 2. Significant hemisphere effects, with left>right FA, were detected at the precentral gyrus (PrG), but not at other levels along the precentral corticospinal pathways. No significant correlations were found between the AQ and the HI scores at p_FWE_ <0.05. When subjects were classified as either right-handers or non-right-handers, no significant handedness effect or hemisphere-by-handedness interactions were observed at a threshold of p_FWE_ <0.05. The TBSS results shown in coronal slices are provided in the supporting documents (Figure S2).

### Skeleton-based ROI analyses

No significant correlation was found between HI and AQ for FA in the three ROIs (i.e., PrG, PLIC, CP) based on Spearman rank correlations. Two-way MANOVA analysis identified a significant effect of hemisphere for mean FA (F_(6,28)_ = 11.37, p<3×10^−6^, Wilks' lambda = 0.34). *Post-hoc* univariate tests of FA values revealed hemispheric effects at the PrG (F_(1,34)_ = 38.37, p<4×10^−7^, partial eta squared = 0.53) and PLIC (F_(1,34)_ = 16.89, p<2×10^−4^, partial eta squared = 0.33), with higher mean FA in the left hemisphere (Table 2). Neither an effect of handedness nor a hemisphere-by-handedness interaction was detected for any skeleton- based ROIs.

**Table 2:** Mean FA values and standard deviations in the ROIs at the precentral gyrus (PrG), the posterior limb of internal capsule (PLIC), the cerebral peduncle (CP), and over the whole precentral corticospinal tract mask (PCST) for right- and non-right-handed chimpanzees based on the TUBE task.

				Handedness on TUBE task			
		All subjects (n = 36)		Right-handed (n = 18)		Non-right handed (n = 18)	
ROI	Hemisphere	Mean	Std.	Mean	Std.	Mean	Std.
**PrG**	left	.5044	.04393	.5013	.04931	.5076	.03900
	right	.4613	.03806	.4587	.04019	.4638	.03678
**PLIC**	left	.5940	.04432	.5946	.05037	.5934	.03881
	right	.5719	.03443	.5734	.03911	.5704	.03011
**CP**	left	.6359	.05990	.6390	.06089	.6328	.06050
	right	.6453	.05193	.6515	.05282	.6391	.05178
**PCST**	left	.4300	.02388	.4321	.02536	.4278	.02282
	right	.4236	.02459	.4229	.02433	.4242	.02553

No significant correlation was found between handedness (HI) and brain asymmetries (AQ) for MD in the three ROIs arrayed along the corticospinal pathway (i.e., PrG, PLIC, CP) based on Spearman rank correlations. Two-way MANOVA analysis identified a significant effect of hemisphere for mean MD (F_(6,28)_ = 4.26, p<0.012, Wilks' lambda = 0.71). *Post-hoc* univariate tests of MD values revealed hemispheric effects at the PrG (F_(1,34)_ = 10.95, p<0.002, partial eta squared = 0.24), with lower mean MD in the left hemisphere (Table 3). Neither an effect of handedness nor a hemisphere-by-handedness interaction was detected for any skeleton- based ROIs.

**Table 3:** Mean MD values (×10^−6^ cm^2^/s) and standard deviations in the ROIs at the precentral gyrus (PrG), the posterior limb of internal capsule (PLIC), the cerebral peduncle (CP), and over the whole precentral corticospinal tract mask (PCST) for right- and non-right-handed chimpanzees based on the TUBE task.

				Handedness on TUBE task			
		All subjects (n = 36)		Right-handed (n = 18)		Non-right handed (n = 18)	
ROI	Hemisphere	Mean	Std.	Mean	Std.	Mean	Std.
**PrG**	left	7.222	0.479	7.239	0.557	7.206	0.402
	right	7.460	0.531	7.544	0.553	7.377	0.509
**PLIC**	left	8.036	0.759	7.895	0.809	8.177	0.700
	right	8.005	0.723	7.880	0.675	8.131	0.767
**CP**	left	9.121	1.058	8.941	1.089	9.302	1.024
	right	9.044	1.068	8.840	1.100	9.249	1.025
**PCST**	left	8.141	0.611	8.062	0.648	8.219	0.580
	right	8.183	0.626	8.125	0.590	8.242	0.672

We further analyzed the axial diffusivity (AD, λ1) and radial diffusivity (RD, (λ2+λ3)/2) to explore the nature of the asymmetries at the corticospinal tract (i.e., axonal changes or myelination). Two-way MANOVA analysis identified a significant effect of hemisphere for mean RD (F_(6,28)_ = 9.17, p<5×10^−5^, Wilks' lambda = 0.46). *Post-hoc* univariate tests of AD values revealed hemispheric effects at the PLIC (F_(1,34)_ = 5.46, p<0.026, partial eta squared = 0.14), with greater AD in the left hemisphere (Table S1). *Post-hoc* univariate tests of RD values revealed a hemispheric effect at the PrG (F_(1,34)_ = 31.74, p<2×10^−6^, partial eta squared = 0.48), with lower RD in the left hemisphere (Table S2). Neither an effect of handedness nor a hemisphere-by-handedness interaction was detected for AD and RD in any skeleton- based ROIs.

### Tract probability map of the precentral corticospinal tracts

**Figure 3:**
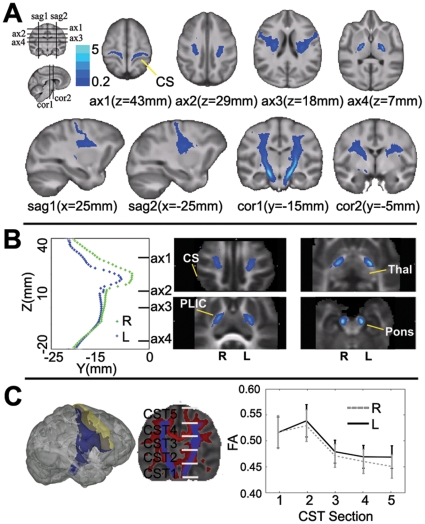
Tract probability map of the precentral corticospinal tracts derived from 36 chimpanzees. **A**: The location of the corticospinal tracts at different levels of the neuraxis is shown in sets of axial (ax), sagittal (sag), and coronal (cor) slices; the locations of these slices are summarized in the figurine on the left. The major part of the tracts with high tract probability streamed through the corona radiata to arch over the putamen, entered the internal capsule, and then passed through the middle portion of the cerebral peduncle down to the level of the seed mask in the basilar pons. **B**: Location of the centers of gravity of tract probability in the left and right hemispheres, depicted graphically and in four axial slices. Voxels that met the >0.2% probability threshold for assignment to the corticospinal tract are marked with blue on the slices. The center of gravity was located more anteriorly in the right hemisphere (red dots in the graph) than in the left hemisphere. Pons: basilar pons; CS: central sulcus; PLIC: posterior limb of internal capsule; Thal: Thalamus. **C**: Plots of hemispheric differences in FA and the 95% confidence intervals in the five equally spaced sections of the corticospinal tract. The left 3D rendering figure shows the lateral view of the precentral corticospinal tracts (thresholded at 0.2%, blue) in an individual T1-weighted image (transparent grey); the coverage of the precentral gyrus mask was outlined in transparent yellow. The middle schematic illustration shows that the precentral corticospinal tract mask (thresholded at 0.2%, blue) were divided into five equally spaced sections ranging from z = 70–150 mm, overlapped on the thresholded mean FA map (FA>0.3, red). The background is the un-thresholded mean FA map of the population.

The tract probability map of the precentral corticospinal tracts from 36 chimpanzees is shown in Fig. 3. In both hemispheres, the reconstructed corticospinal tracts with high tract probability extended from the lateral part of the PrG into the corona radiata, with separate branches also originating from the ventral PrG. The main part of the tracts with high tract probability streamed through the corona radiata to arch over the putamen and then entered the internal capsule, passing through the middle portion of the cerebral peduncle down to the level of the seed mask in the basilar pons. Starting approximately from the level of the thalamus (z = 0 mm) and above, the gravity center of the right precentral corticospinal tract for the sample was located more anteriorly than that of the left side (Figs. 3A & B). The magnitude of this anterior-posterior positional difference was approximately 3 mm.

Tractography with the seed masks placed at a more inferior level of the basilar pons was performed on four chimpanzees and the results were similar as those with the seed masks in the middle level of the basilar pons (Fig. S3).

### Tract-based ROI analyses

No significant correlation was found between handedness (HI) and brain asymmetries (AQ) for FA in the tract-based ROIs (i.e., the mean FA from within the whole precentral corticospinal tract and from the five equally spaced sectors) according to Spearman rank correlations. Two-way MANOVA analysis identified a significant effect of hemisphere on mean FA (F_(6,28)_ = 58.83, p<1×10^−14^, Wilks' lambda = 0.069). *Post-hoc* univariate tests of FA values revealed hemispheric effects for the mean FA from within the whole corticospinal tract (F_(1,34)_ = 6.63, p<0.015, partial eta squared = 0.163), at CST2 (F_(6,28)_ = 5.17, p<0.029, partial eta squared  = 0.13), at CST3(F_(6,28)_ = 4.84, p<0.035, partial eta squared  = 0.13), at CST4 (F_(6,28)_ = 6.78, p<0.014, partial eta squared  = 0.17), and at CST5 (F_(6,28)_ = 24.69, p<1×10^−5^, partial Eta Squared  = 0.42) (Fig. 3C), with higher FA in the left hemisphere. A hemispheric effect on the corticospinal tract volume was also detected (F_(6,28)_ = 9.09, p<0.005, partial Eta Squared  = 0.21), with larger corticospinal tract volume in the right hemisphere than the left. Neither an effect of handedness nor a hemisphere-by-handedness interaction was detected for tract-based ROIs.

No significant correlation was found between handedness (HI) and brain asymmetries (AQ) for MD in the tract-based ROIs according to Spearman rank correlations. Two-way MANOVA analysis identified a significant effect of hemisphere on mean MD (F_(6,28)_ = 15.59, p<6×10^−8^, Wilks' lambda = 0.24). *Post-hoc* univariate tests of MD values revealed hemispheric effects for the mean MD at CST1 (F_(6,28)_ = 19.14, p<1×10^−4^, partial eta squared  = 0.36) with higher MD in the left hemisphere, at CST4(F_(6,28)_ = 37.87, p<5×10^−7^, partial eta squared  = 0.53) and at CST5 (F_(6,28)_ = 12.39, p<0.001, partial eta squared  = 0.27, with lower MD in the left hemisphere. Neither an effect of handedness nor a hemisphere-by-handedness interaction was detected for tract-based ROIs. No effect of handedness or hemisphere, or the interaction of the two, was observed for mean AD, RD within the tract-based ROIs.

### Depth of the central sulcus

**Figure 4:**
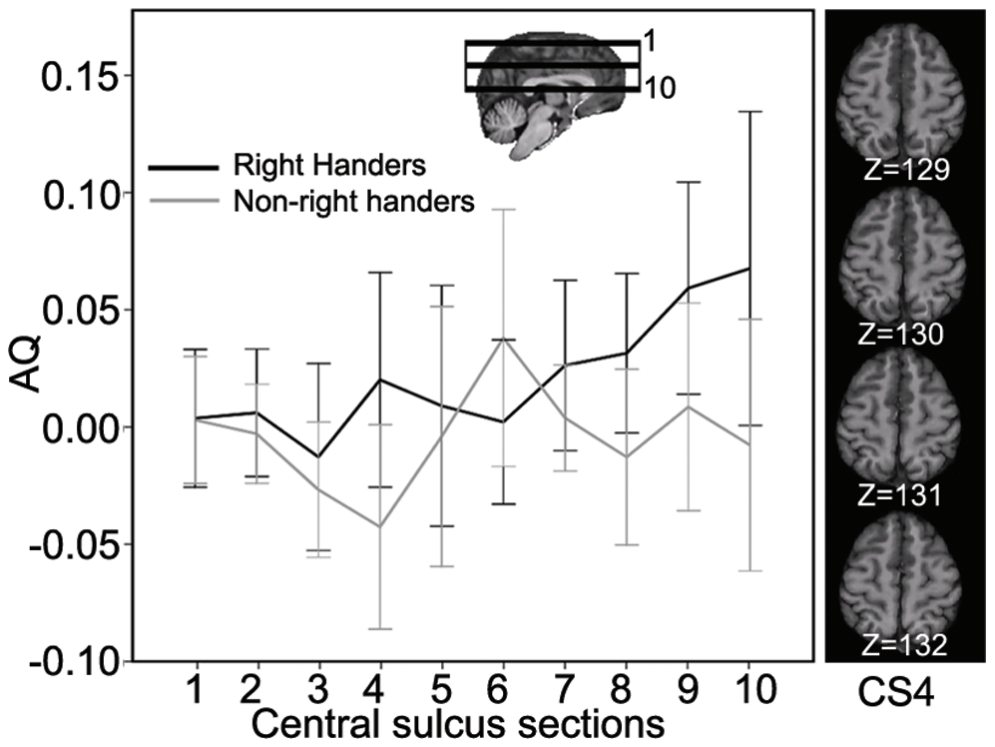
Mean asymmetry quotients of the depth of the CS and the 95% confidence interval at 10 levels, numbered from superior (CS1) to inferior (CS10), plotted separately for chimpanzees with different handedness. The four representative axial slices from one subject corresponding to “hand knob” (CS4) are shown on the right side.

A hemisphere-by-handedness interaction was detected at CS10 (F_(1,34)_ = 5.202, p<0.029, partial eta squared  = 0.133) and CS4 (F_(1,34)_ = 4.810,p<0.035, partial eta squared  = 0.124) (see Fig. 4), with a deeper central sulcus in these sectors in the left hemisphere for right-handers than for non-right-handers. No handedness effect or hemisphere-by-handedness interaction was found at any other level of the CS or for the total depth of the central sulcus.

Concerning the relationship between CS depth and corticospinal FA and MD, we found no significant correlation between the AQ of the depth of the central sulcus and the AQ of the mean FA or MD of the precentral corticospinal tract mask.

## Discussion

This is the first study evaluating the asymmetries of chimpanzees' precentral corticospinal system and their relationship to handedness using diffusion MRI. The results of this study demonstrate interhemispheric asymmetries in the precentral corticospinal system of chimpanzees that involve both microstructural (as reflected by FA and MD) as well as macrostructural (as reflected by anatomical location) differences. No handedness effect or hemisphere-by-handedness interaction was detected in any of the diffusion MRI-derived metrics. The depth of the central sulcus, which was also measured to assess gross asymmetries in the primary motor cortex, was found to be associated with handedness in the present study, consistent with previous studies of humans and of chimps. However, we found no significant correlation between the asymmetries of central sulcus depth and FA or MD in the precentral corticospinal system.

### Hemispheric asymmetries at micro- and macrostructural levels

Our results indicate that chimpanzees show a leftward bias (left>right) in FA of the corticospinal tracts, measured over the entire tract (using the track-based ROI method) and at the level of the precentral gyrus and posterior limb of internal capsule (using the TBSS method), which are largely paralleled by a rightward bias (right>left) in MD in the subsequent skeleton-based and tract-based ROI analysis. Our results in chimpanzees are generally consistent with those reported in humans, which show leftward biases in FA in the corticospinal tracts [43]. In addition, the use of probabilistic fiber tracking made it possible for us to detect asymmetries in the locations of the chimpanzee corticospinal tracts in the cerebral white matter, and we found that the right precentral corticospinal is centered more anteriorly than that of the left. These observations in chimpanzees are also generally consistent with those reported in humans [67], [72], [73]. For example, Rademacher *et al.* generated a variability map of the corticospinal tract in humans by registering the myelin-stained pyramidal tracts from the 10 postmortem brains to Talairach coordinates and found that the right pyramidal tract was centered more anteriorly than the left.

In an attempt to explore the nature of the asymmetries observed in our chimpanzee population, we analyzed the asymmetries of the corticospinal tract indexed by AD and RD, where have been proposed to serve as markers for differentiating degrees of myelination and changes in axons [74], [75], [76], [77]. As we have observed no hemispheric asymmetry in the AD and RD within the tract-based ROIs, and only regional asymmetries in the two indices in the skeleton-based ROI analyses (greater AD in the left PLIC and lower RD in the left PrG), we believe that we are currently not at the stage to conclude what mechanism (i.e., changes in axons or in degrees of myelination in two hemispheres) underlies the microstructural asymmetries of chimpanzees' corticospinal tract.

Even though the interhemispheric asymmetries in the corticospinal tracts detected in chimpanzees in this study are qualitatively similar to those reported in humans, with a leftward bias in FA and a right-anterior shift in the anatomical position of the tract, it is still possible that human corticospinal tracts are more asymmetrical than those of chimpanzees. Direct comparisons using the same methodology on the two species are necessary to characterize possible quantitative differences and are currently being pursued by our group.

### Relationships between hemispheric FA, MD asymmetry and handedness

Despite the hemispheric leftward bias in FA and MD detected in the chimpanzee precentral corticospinal system, we detected neither a relationship between FA or MD and handedness nor any hemisphere-by-handedness interaction in either the voxelwise or the ROI analyses at the levels examined in the present study, which included the precentral gyrus white matter, the posterior limb of the internal capsule, the cerebral peduncle, and the whole precentral corticospinal tract. These results are consistent with two studies in humans that reported no hemisphere-by-handedness interactions in the CST at the level of the PLIC [43] and over the entire CST [40]. However, Buchel and colleagues, in humans, have reported higher FA in the white matter of the PrG in the left hemisphere of right handers and in the right hemisphere of left handers [29]; that is, a hemisphere-by-handedness interaction. Additional studies of humans with comparable methodologies and samples are necessary to resolve these apparently discrepant results.

### Relationship between hemispheric asymmetry in the depth of the central sulcus and handedness

Interestingly, in contrast to studies of diffusion MR data, previous studies using gross anatomical markers, such as the depth of the central sulcus and/or volume of the hand knob, consistently report a hemisphere-by-handedness interaction. For example, in studies measuring the depth of the central sulcus, hemisphere-by-handedness interactions have been reported in multiple human studies [27], [28], [78], [79] as well as in one previous chimpanzee study [46]. Similarly, in the present study, we found significant correlations between the asymmetry of the depth of the central sulcus (as indexed by AQ) and handedness (indexed by HI) detected at several levels of the central sulcus, including the level corresponding to the “hand knob” (CS4) (see Fig. 4). However, no significant relationship was found between the AQ of central sulcus depth and the AQ of FA or MD at any level of the precentral corticospinal system. These observations suggest—both for chimpanzees and for humans—that the asymmetries of central sulcus depth and of corticospinal FA and MD are largely unrelated, with only the central sulcus depth strongly related to handedness. If asymmetries of the central sulcus depth reflect differences in the precentral gray matter volume in the left and right hemispheres, as seems likely, then hand dominance is related more strongly to interhemispheric differences in cortical gray matter volume than to interhemispheric differences in the microstructural features of the corticospinal tract white matter indexed by FA/MD, such as axonal packing density, myelination, and fiber coherence [80]. We cannot rule out the possibility, however, that the lack of correlation between the hemispheric asymmetries of the CST indexed by diffusion MR data and depth of the central sulcus, as well as handedness, is a type II error. That is, there could be microstructural differences in the white matter related to handedness too small to be detected without improvements in imaging methodology or the use of larger sample sizes. Similarly, we might also have failed to detect a handedness effect due to the fact that there are differences in aspects of microstructure not reflected by FA.

If interhemispheric asymmetries reflected in FA and MD in the precentral corticospinal system of chimpanzees (and perhaps also humans) are not related to hand preference, what is their functional significance? One possibility is that the hemispheric bias in FA and MD reflects the existence in chimpanzees of a left hemisphere network for action planning, which includes parietal, premotor, prefrontal and posterior temporal cortices and is independent of the hand involved in producing the movements and handedness, as has been proposed for humans [81], [82]. Recent observations on complex tool-use skills on chimpanzees [83], [84] indicate that a similar left cerebral hemisphere network for action planning may also exist in chimpanzees that is independent of handedness. The higher FA and lower MD in the left corticospinal tract observed here may thus reflect increased myelination and/or higher fiber packing density on the left, supporting the action planning network. Another possible explanation for the hemispheric asymmetries in FA and MD could be that, like humans, the two hemispheres in chimpanzees follow somewhat divergent developmental trajectories, with a variety of physical asymmetries emerging in different stages of life [85], [86]. The higher FA (lower MD) observed in the left precentral corticospinal tracts might be a result of differential growth of the white matter in the two hemispheres. Nevertheless, direct evidence that links the corticospinal tract asymmetries reflected by diffusion MRI to developmental differences in histology is not available, and further investigations are warranted.

### Methodological issues

Several methodological limitations should be considered in interpreting the results in the present study. First, only female chimpanzees were included here, while at least one diffusion study in humans has reported a main effect of sex on FA asymmetry of corticospinal tracts [43]. Although sex effects in chimpanzees were not found for volumetric asymmetry of motor hand areas [45], to our knowledge no study has investigated this effect using diffusion MRI. This limitation also applies in the investigation of the association between interhemispheric asymmetry and handedness [28], [87] and might explains the discrepancy between our results and those in one human diffusion MRI study including both male and female subjects [29]. Therefore, it is possible that, when both males and females are included, the patterns of asymmetry in chimpanzees' corticospinal system as well as its relationship with handedness might differ.

In tracking the corticospinal tract, we used a seed mask at the middle level of the basilar pons. Very similar approaches have been used by others to delineate the corticospinal tracts in humans [88], [89], [90], [91], [92], and subcortical motor-evoked potential studies in humans confirmed that the fiber system delineated in this way includes the corticospinal tract [89]. This approach is not ideal, however, because the white matter of the basilar pons includes not only corticospinal fibers but also a large number of corticopontine fibers [88], [93], [94] and corticobulbar fibers [94], as well as cortico-olivary and cortico-reticular fibers. Since the corticobulbar system can be considered a brainstem extension of the corticospinal system, and the cortico-olivary and cortico-reticular systems are likely small, our main concern is that the tracts we delineated may have included a substantial number of fibers from the large corticopontine tract in addition to corticospinal fibers. Corticopontine fibers could be largely excluded by placing seed masks caudal to the pons; ideally, the masks would be drawn at the decussation of the pyramids. There are compelling practical reasons, however, for drawing the seed masks at the level of the middle pons and not lower in the brain stem. In our study (and probably in the human studies cited above, in which fibers were tracked in similar fashion) the scans did not always extend far enough inferiorly to include the decussation, and even when they did, the image quality in the diffusion MRI was often poor at these inferior levels, owing to large susceptibility artifact and other physiology-related artifacts common at the base of the brain. Drawing seed masks at the middle level of the basilar pontine white matter mitigated these problems, and is consistent with procedures used in most of the humans studies to which we want to compare our results in chimpanzees [88], [91], [95]. To determine whether the mid-pontine seed mask we used for reconstructing corticospinal tracts in our 36 chimpanzees also intercepted a large number of corticopontine fibers, we repeated our analysis in 4 of these chimpanzees with good image quality at inferior levels, drawing seed masks at much lower levels of the brainstem (inferior to the levels at which corticopontine fibers terminate in the pontine gray), and we obtained tracts almost identical to those with the seed mask at the middle level of the pons (see Fig. S3). This suggests that the fiber tracts we delineated using seed masks at middle levels of the basilar pons consisted very largely of corticospinal fibers and did not include a substantial number of corticopontine fibers.

We used a tract-based ROI method as an alternative to defining white-matter ROIs or using voxelwise TBSS analysis. This approach has been previously employed both in clinical conditions [96], [97] and in basic neuroscience [77], [98], [99] and has several potential advantages over TBSS alone and ROI analyses. First, DTI-derived metrics (FA and MD) derived from within the tractography-defined corticospinal pathway should be highly relevant to motor functions. Second, voxelwise analysis is sensitive to asymmetric white-matter distribution only when the differences between left and right corticospinal tracts are located in approximately the same regions across subjects. It can produce false negatives, for example, when DTI-derived metrics changes occur along a specific tract, but the exact location where the change is most pronounced along the tract differs across subjects or hemispheres. It was therefore useful to employ an alternative, specifically, the mean FA and MD from within the entire precentral corticospinal tract delineated by probabilistic tractography to investigate hemispheric and handedness effects.

Our results revealed that the chimpanzee precentral corticospinal system displays a clear hemispheric asymmetry in FA and MD, and in its anatomical position. Nonetheless, handedness is not correlated with these hemispheric asymmetries in FA and MD, although it is correlated with the asymmetries of the central sulcus depth. Our data raise the possibility that the left-right asymmetries in FA and MD and tract location observed using diffusion MRI, in chimpanzees and in humans, are not directly related to handedness. The evolutionary origin and functional consequences of these cerebral asymmetries remain to be explained.

## Supporting Information

Figure S1 The 60 diffusion gradient directions extracted from the diffusion MRI sequence source code plotted on a transparent half sphere. The exact orientation of the gradient table for each chimpanzee differs due to various positioning angles applied in each scan.
(7.23 MB TIF)

Figure S2 TBSS analysis of the relationship between hemisphere and FA in the precentral corticospinal system. The figurine on the left shows the positions of the six coronal slices. FA statistical maps are superimposed on the mean FA skeletons, voxels shaded green are those for which FA exceeds threshold (FA>0.3). The colors superimposed on these maps indicate FA asymmetries, with red denoting left>right FA and blue denoting right>left FA. The red and blue pixels are enlarged for emphasis. Extensive left>right FA was found at the precentral gyrus. No right>left FA that is relevant to the corticospinal system was detected.
(2.83 MB TIF)

Figure S3 Comparison of the two probability maps of the corticospinal tracts with the seed masks located at different levels of basilar pons. (A) The positions of the seed masks for two different runs of tracking corticospinal tract; For one run, the seed mask was drawn at the middle level (S1) and for another, it was at more inferior level (S2) of the basilar pons; (B) Almost identical results of the two probability maps were obtained, with the seed masks located at the two different levels of basilar pons. All the other waypoint and cortical target masks were identical for generating the two probability maps.
(1.98 MB TIF)

Table S1 Mean AD values (10E-5 cm2/s) and standard deviations in the ROIs at the precentral gyrus (PrG), the posterior limb of internal capsule (PLIC), the cerebral peduncle (CP), and over the whole precentral corticospinal tract mask (PCST) for right- and non-right-handed chimpanzees based on the TUBE task.
(0.03 MB DOC)

Table S2 Mean RD values (10E-5 cm2/s) and standard deviations in the ROIs at the precentral gyrus (PrG), the posterior limb of internal capsule (PLIC), the cerebral peduncle (CP), and over the whole precentral corticospinal tract mask (PCST) for right- and non-right-handed chimpanzees based on the TUBE task.
(0.03 MB DOC)
