# Supplementary figures and images for: Chimpanzee (Pan troglodytes) Precentral Corticospinal System Asymmetry and Handedness: A Diffusion Magnetic Resonance Imaging Study
Source: PLoS One. 2010 Sep 21;5(9):e12886. doi: 10.1371/journal.pone.0012886 (PMC2943482; doi:10.1371/journal.pone.0012886)

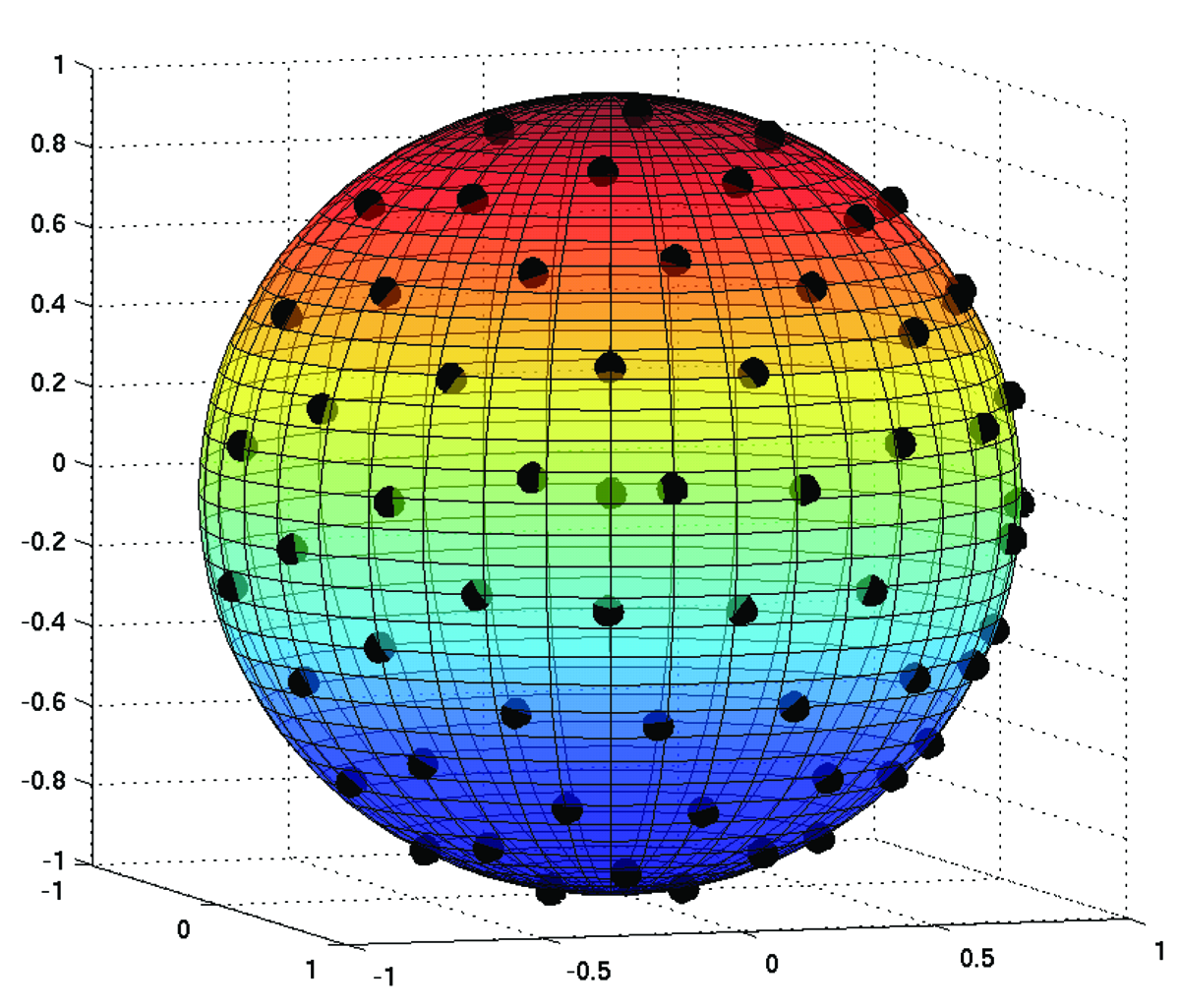

Supplement: Figure S1 — The 60 diffusion gradient directions extracted from the diffusion MRI sequence source code plotted on a transparent half sphere. The exact orientation of the gradient table for each chimpanzee differs due to various positioning angles applied in each scan. (7.23 MB TIF) [file pone.0012886.s001.tif]

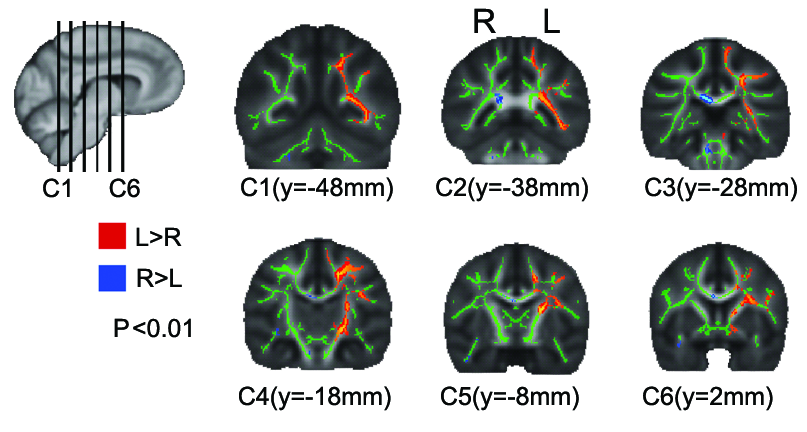

Supplement: Figure S2 — TBSS analysis of the relationship between hemisphere and FA in the precentral corticospinal system. The figurine on the left shows the positions of the six coronal slices. FA statistical maps are superimposed on the mean FA skeletons, voxels shaded green are those for which FA exceeds threshold (FA>0.3). The colors superimposed on these maps indicate FA asymmetries, with red denoting left>right FA and blue denoting right>left FA. The red and blue pixels are enlarged for emphasis. Extensive left>right FA was found at the precentral gyrus. No right>left FA that is relevant to the corticospinal system was detected. (2.83 MB TIF) [file pone.0012886.s002.tif]

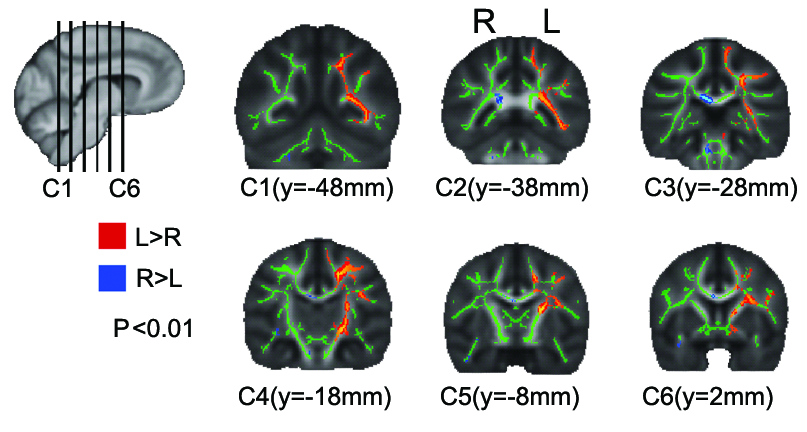

Supplement: Figure S3 — Comparison of the two probability maps of the corticospinal tracts with the seed masks located at different levels of basilar pons. (A) The positions of the seed masks for two different runs of tracking corticospinal tract; For one run, the seed mask was drawn at the middle level (S1) and for another, it was at more inferior level (S2) of the basilar pons; (B) Almost identical results of the two probability maps were obtained, with the seed masks located at the two different levels of basilar pons. All the other waypoint and cortical target masks were identical for generating the two probability maps. (1.98 MB TIF) [file pone.0012886.s003.tif]
