# Supplementary material for: Chimpanzee (Pan troglodytes) Precentral Corticospinal System Asymmetry and Handedness: A Diffusion Magnetic Resonance Imaging Study
Source: PLoS One. 2010 Sep 21;5(9):e12886. doi: 10.1371/journal.pone.0012886 (PMC2943482; doi:10.1371/journal.pone.0012886)
Supplement: Table S2 — Mean RD values (10E-5 cm2/s) and standard deviations in the ROIs at the precentral gyrus (PrG), the posterior limb of internal capsule (PLIC), the cerebral peduncle (CP), and over the whole precentral corticospinal tract mask (PCST) for right- and non-right-handed chimpanzees based on the TUBE task. (0.03 MB DOC) [file pone.0012886.s005.doc]

Supplementary Table 2. Mean RD values (×10^-5^ cm^2^/s) and standard deviations in the ROIs at the precentral gyrus (PrG), the posterior limb of internal capsule (PLIC), the cerebral peduncle (CP), and over the whole precentral corticospinal tract mask (PCST) for right- and non-right-handed chimpanzees based on the TUBE task.

|  |  |  | | Handedness on TUBE task | | | |
| --- | --- | --- | --- | --- | --- | --- | --- |
|  |  | All subjects (n = 36) | | Right-handed (n = 18) | | Non-right handed (n=18) | |
| **ROI** | **Hemisphere** | **Mean** | **Std.** | **Mean** | **Std.** | **Mean** | **Std.** |
| **PrG** | left | 0.507 | 0.051 | 0.512 | 0.059 | 0.503 | 0.043 |
|  | right | 0.550 | 0.058 | 0.556 | 0.059 | 0.544 | 0.058 |
| **PLIC** | left | 0.515 | 0.077 | 0.504 | 0.084 | 0.526 | 0.070 |
|  | right | 0.528 | 0.067 | 0.517 | 0.064 | 0.540 | 0.069 |
| **CP** | left | 0.530 | 0.108 | 0.517 | 0.121 | 0.542 | 0.096 |
|  | right | 0.518 | 0.097 | 0.501 | 0.103 | 0.534 | 0.091 |
| **PCST** | left | 0.617 | 0.058 | 0.611 | 0.061 | 0.624 | 0.055 |
|  | right | 0.623 | 0.058 | 0.619 | 0.056 | 0.628 | 0.063 |
